# Di-μ-hydroxido-bis­({2,2′-[propane-1,3-diylbis(nitrilo­methyl­idyne)]diphenolato}iron(III)) dimethyl­formamide disolvate

**DOI:** 10.1107/S1600536809005091

**Published:** 2009-02-21

**Authors:** Qingyun Liu, Shuying Pan, Dongmei Wang

**Affiliations:** a School of Chemical & Environmental Engineering, Shandong University of Science and Technology, Qingdao 266510, People’s Republic of China; b Soil and Fertilizer General Monitoring Station of Shandong Province, Jinan 250100, People’s Republic of China

## Abstract

The structure of the title compound, [Fe_2_(C_17_H_16_N_2_O_2_)_2_(OH)_2_]·2C_3_H_7_N, consists of centrosymmetric dimeric units in which crystallographically equivalent Fe^III^ ions are doubly bridged by hydroxide groups. Each Fe^III^ center in the complex has a six-coordinated distorted *cis*-FeN_2_O_4_ octa­hedral geometry.

## Related literature

For background to the use of Schiff base ligands in the assembly of hydroxo-, alkoxo- or phenoxo-bridged clusters and polymers, see: Chen *et al.* (2006 ▶); Koizumi *et al.* (2005 ▶); Ni & Wang (2007 ▶). For the use of H_2_salpn as a flexible ligand, see: Ni *et al.* (2005 ▶); Si *et al.* (2002 ▶).
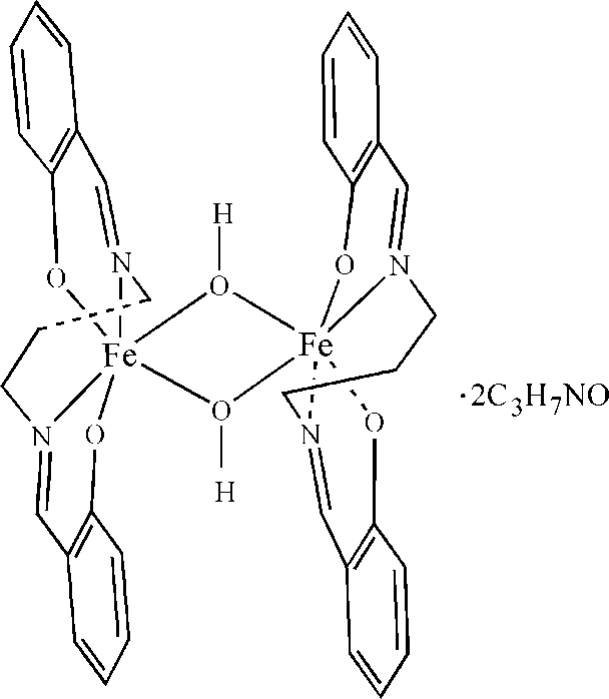


## Experimental

### 

#### Crystal data

[Fe_2_(C_17_H_16_N_2_O_2_)_2_(OH)_2_]·2C_3_H_7_N
*M* *_r_* = 852.54
Monoclinic, 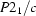

*a* = 10.768 (2) Å
*b* = 10.136 (2) Å
*c* = 17.540 (4) Å
β = 101.27 (3)°
*V* = 1877.5 (7) Å^3^
*Z* = 2
Mo *K*α radiation
μ = 0.84 mm^−1^
*T* = 293 K
0.21 × 0.15 × 0.12 mm

#### Data collection

Bruker APEXII CCD area-detector diffractometer
Absorption correction: multi-scan (*SADABS*; Sheldrick, 2003 ▶) *T*_min_ = 0.862, *T*_max_ = 0.908
10664 measured reflections
3221 independent reflections
2809 reflections with *I* > 2σ(*I*)
*R*_int_ = 0.038

#### Refinement

*R*[*F* ^2^ > 2σ(*F* ^2^)] = 0.046
*wR*(*F* ^2^) = 0.143
*S* = 1.04
3221 reflections
253 parameters
H-atom parameters constrained
Δρ_max_ = 0.48 e Å^−3^
Δρ_min_ = −0.65 e Å^−3^

### 

Data collection: *APEX2* (Bruker, 2004 ▶); cell refinement: *SAINT-Plus* (Bruker, 2001 ▶); data reduction: *SAINT-Plus*; program(s) used to solve structure: *SHELXS97* (Sheldrick, 2008 ▶); program(s) used to refine structure: *SHELXL97* (Sheldrick, 2008 ▶); molecular graphics: *XP* in *SHELXTL* (Sheldrick, 2008 ▶); software used to prepare material for publication: *XP* in *SHELXTL*.

## Supplementary Material

Crystal structure: contains datablocks global, I. DOI: 10.1107/S1600536809005091/hg2478sup1.cif

Structure factors: contains datablocks I. DOI: 10.1107/S1600536809005091/hg2478Isup2.hkl

Additional supplementary materials:  crystallographic information; 3D view; checkCIF report

## Figures and Tables

**Fig. 1.**
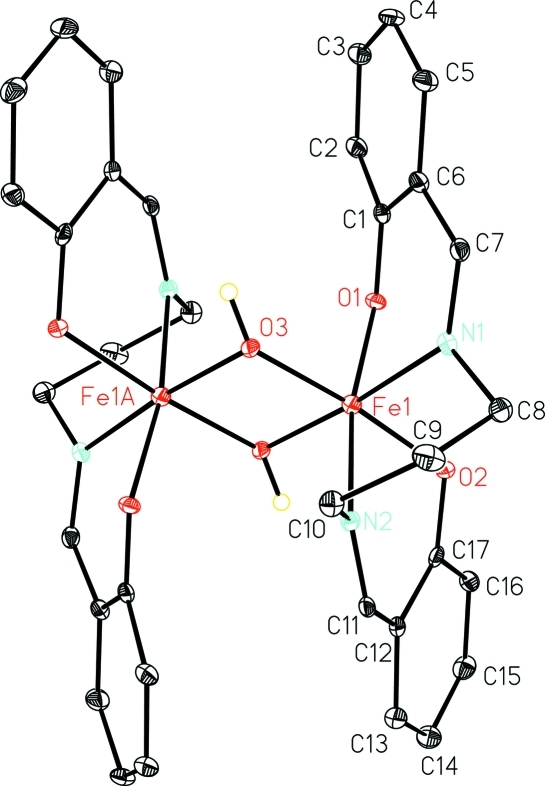
A view of (I) with the unique atom-labelling scheme. Displacement ellipsoids are drawn at the 30% probability level.

## supplementary crystallographic information

### Comment

Recently, the the Schiff base ligands, especially the relative flexible symmetrical or unsymmetrical Schiff base ligands and their hydrogenerated derivatives have been widely employed to assembly hydroxo-, alkoxo- or phenoxo-bridged clusters and polymers with novel topological structures and interesting magnetic, catalysis and photochemical properties (Chen *et al.*, 2006; Koizumi *et al.*, 2005; Ni & Wang, 2007). Among Schiff base ligands, H_2_salpn is a special symmetrical tetradentate Schiff base which can be used as rigid ligand located on the equatorial plane of central metal ion. However, sometimes it also exhibits characteristics of a flexible ligand situated at the terminal of metal ions like a cap. To date, several examples in which H_2_salpn is used as flexible ligand have been synthesized (Ni *et al.*, 2005; Si *et al.*, 2002) We recently synthesized a doubly hydroxo-bridged Fe^III^ dimeric complex where Schiff base H_2_salpn exist as flexible terminal ligand with two free DMF solvate molecules, Herein, we reported the synthesis and crystal structure of the complex [Fe^III^(salpn)OH]_2_.2DMF (I).

The geometry and labeling scheme for the crystal structure of [Fe^III^(salpn)OH]_2_.2DMF (I) is depicted in Figure 1. The structure of I consists of centrosymmetric dimer units in which crystallographically equivalent iron(III) ions are doubly bridged by hydroxo groups. The ligand with N_2_O_2_ donating atoms occupies four coordination sites of the Fe^III^ ion while the two remaining ones are filled by the bridging hydroxo groups. The Fe^III^ ions have a slightly distorted-octahedral coordination geometry. The four coordination atoms from salpn^2-^ are not in a plane for distortion of carbon linkage and the requirement of coordination of hydroxo-bridging groups from the same side to take *cis*-disposition. The two Fe—O bond distances in the bridging unit are 1.8158 (18) and 1.8172 (18) Å, respectively.

### Experimental

Ligand H_2_salpn (0.5 mmol) was added to a solution of Fe(ClO_4_)_2_.4H_2_O (0.5 mmol) in MeOH and DMF mixing solvent (1/1, *v*/*v*). The mixture was stirred for about four hours, and then filtered. The filrate was evaporated in room temperature for two days yielding red brown single-crystals. Yield: 30%. Elemental analysis [found (calculated)] for C_40_H_48_Fe_2_N_6_O_8_: C 56.08 (56.35), H 5.45 (5.67), N 9.80 (9.86%).

### Refinement

H atoms bound to C and O atoms were visible in difference maps and were placed using the HFIX commands in *SHELXL-97*. H atoms were placed in calculated positions and were included in the refinement in the riding-model approximation, All H atoms were allowed for as riding atoms (C—H 0.96 Å or C—H 0.93 Å, and O—H 0.85 Å) with the constraint *U*_iso_(H) = 1.5*U*_eq_(methyl carrier), 1.5*U*_eq_(O) and *U*_iso_(H) = 1.2*U*eq(C) for all other H atoms..

### Crystal data

**Table:**

[Fe_2_(C_17_H_16_N_2_O_2_)_2_(OH)_2_]·2C_3_H_7_N	*F*(000) = 892
*M**_r_* = 852.54	*D*_x_ = 1.508 Mg m^−^^3^
Monoclinic, *P*2_1_/*c*	Mo *K*α radiation, λ = 0.71073 Å
Hall symbol: -P 2ybc	Cell parameters from 221 reflections
*a* = 10.768 (2) Å	θ = 3.1–25.0°
*b* = 10.136 (2) Å	µ = 0.84 mm^−^^1^
*c* = 17.540 (4) Å	*T* = 293 K
β = 101.27 (3)°	Block, brown
*V* = 1877.5 (7) Å^3^	0.21 × 0.15 × 0.12 mm
*Z* = 2	

### Data collection

**Table:**

Bruker APEXII CCD area-detector diffractometer	3221 independent reflections
Radiation source: fine-focus sealed tube	2809 reflections with *I* > 2σ(*I*)
graphite	*R*_int_ = 0.038
φ and ω scans	θ_max_ = 25.0°, θ_min_ = 3.1°
Absorption correction: multi-scan (*SADABS*; Sheldrick, 2003)	*h* = −12→12
*T*_min_ = 0.862, *T*_max_ = 0.908	*k* = −12→11
10664 measured reflections	*l* = −20→20

### Refinement

**Table:**

Refinement on *F*^2^	Primary atom site location: structure-invariant direct methods
Least-squares matrix: full	Secondary atom site location: difference Fourier map
*R*[*F*^2^ > 2σ(*F*^2^)] = 0.046	Hydrogen site location: inferred from neighbouring sites
*wR*(*F*^2^) = 0.143	H-atom parameters constrained
*S* = 1.04	*w* = 1/[σ^2^(*F*_o_^2^) + (0.0957*P*)^2^ + 1.7319*P*] where *P* = (*F*_o_^2^ + 2*F*_c_^2^)/3
3221 reflections	(Δ/σ)_max_ = 0.001
253 parameters	Δρ_max_ = 0.48 e Å^−^^3^
0 restraints	Δρ_min_ = −0.65 e Å^−^^3^

### Special details

**Table:**

Geometry. All e.s.d.'s (except the e.s.d. in the dihedral angle between two l.s. planes) are estimated using the full covariance matrix. The cell e.s.d.'s are taken into account individually in the estimation of e.s.d.'s in distances, angles and torsion angles; correlations between e.s.d.'s in cell parameters are only used when they are defined by crystal symmetry. An approximate (isotropic) treatment of cell e.s.d.'s is used for estimating e.s.d.'s involving l.s. planes.
Refinement. Refinement of *F*^2^ against ALL reflections. The weighted *R*-factor *wR* and goodness of fit *S* are based on *F*^2^, conventional *R*-factors *R* are based on *F*, with *F* set to zero for negative *F*^2^. The threshold expression of *F*^2^ > σ(*F*^2^) is used only for calculating *R*-factors(gt) *etc*. and is not relevant to the choice of reflections for refinement. *R*-factors based on *F*^2^ are statistically about twice as large as those based on *F*, and *R*- factors based on ALL data will be even larger.

### Fractional atomic coordinates and isotropic or equivalent isotropic displacement parameters (Å^2^)

**Table:**

	*x*	*y*	*z*	*U*_iso_*/*U*_eq_	
Fe1	0.50501 (4)	0.37734 (4)	0.03284 (2)	0.01386 (19)	
O1	0.54651 (17)	0.43569 (19)	0.13793 (11)	0.0140 (4)	
O2	0.63819 (18)	0.24760 (19)	0.05879 (11)	0.0148 (4)	
O3	0.39002 (17)	0.50795 (18)	0.00288 (10)	0.0127 (4)	
H3B	0.3390	0.5576	0.0209	0.015*	
O4	0.9440 (2)	0.6587 (3)	0.27864 (14)	0.0360 (6)	
N1	0.3654 (2)	0.2624 (2)	0.06536 (13)	0.0135 (5)	
N2	0.4579 (2)	0.2834 (2)	−0.07001 (13)	0.0128 (5)	
N3	0.9331 (2)	0.5320 (3)	0.16973 (15)	0.0251 (6)	
C1	0.4628 (3)	0.4666 (3)	0.18093 (15)	0.0131 (6)	
C2	0.4952 (3)	0.5623 (3)	0.24001 (17)	0.0186 (6)	
H2A	0.5754	0.6002	0.2486	0.022*	
C3	0.4098 (3)	0.6008 (3)	0.28532 (18)	0.0216 (7)	
H3A	0.4322	0.6665	0.3225	0.026*	
C4	0.2905 (3)	0.5423 (3)	0.27608 (17)	0.0213 (7)	
H4A	0.2329	0.5701	0.3059	0.026*	
C5	0.2586 (3)	0.4420 (3)	0.22176 (17)	0.0206 (7)	
H5A	0.1811	0.3993	0.2173	0.025*	
C6	0.3431 (3)	0.4043 (3)	0.17312 (17)	0.0152 (6)	
C7	0.3077 (3)	0.2952 (3)	0.12009 (16)	0.0156 (6)	
H7A	0.2379	0.2451	0.1261	0.019*	
C8	0.3228 (3)	0.1439 (3)	0.01922 (18)	0.0190 (6)	
H8A	0.2676	0.0928	0.0454	0.023*	
H8B	0.3957	0.0897	0.0156	0.023*	
C9	0.2526 (3)	0.1786 (3)	−0.06223 (17)	0.0231 (7)	
H9A	0.1701	0.2139	−0.0589	0.028*	
H9B	0.2391	0.0985	−0.0930	0.028*	
C10	0.3225 (3)	0.2798 (3)	−0.10456 (17)	0.0170 (6)	
H10A	0.3116	0.2562	−0.1591	0.020*	
H10B	0.2861	0.3666	−0.1013	0.020*	
C11	0.5369 (3)	0.2250 (3)	−0.10447 (16)	0.0146 (6)	
H11A	0.5069	0.1928	−0.1543	0.018*	
C12	0.6696 (3)	0.2055 (3)	−0.07143 (17)	0.0149 (6)	
C13	0.7523 (3)	0.1622 (3)	−0.11903 (18)	0.0199 (6)	
H13A	0.7222	0.1542	−0.1723	0.024*	
C14	0.8760 (3)	0.1314 (3)	−0.0891 (2)	0.0236 (7)	
H14A	0.9297	0.1035	−0.1215	0.028*	
C15	0.9204 (3)	0.1426 (3)	−0.00837 (19)	0.0209 (7)	
H15A	1.0046	0.1232	0.0124	0.025*	
C16	0.8409 (3)	0.1821 (3)	0.04073 (17)	0.0172 (6)	
H16A	0.8718	0.1870	0.0940	0.021*	
C17	0.7135 (3)	0.2150 (3)	0.01030 (17)	0.0146 (6)	
C18	0.9718 (3)	0.6354 (3)	0.2146 (2)	0.0293 (8)	
H18A	1.0243	0.6957	0.1964	0.035*	
C19	0.8528 (3)	0.4313 (3)	0.1945 (2)	0.0289 (7)	
H19A	0.8366	0.4537	0.2448	0.043*	
H19B	0.8948	0.3474	0.1971	0.043*	
H19C	0.7741	0.4264	0.1578	0.043*	
C20	0.9676 (4)	0.5132 (4)	0.0947 (2)	0.0354 (8)	
H20A	1.0208	0.5846	0.0847	0.053*	
H20B	0.8924	0.5111	0.0549	0.053*	
H20C	1.0125	0.4314	0.0946	0.053*	

### Atomic displacement parameters (Å^2^)

**Table:**

	*U*^11^	*U*^22^	*U*^33^	*U*^12^	*U*^13^	*U*^23^
Fe1	0.0147 (3)	0.0133 (3)	0.0142 (3)	0.00014 (14)	0.00425 (18)	0.00012 (15)
O1	0.0146 (10)	0.0165 (10)	0.0118 (10)	0.0006 (7)	0.0051 (8)	−0.0002 (8)
O2	0.0164 (10)	0.0152 (10)	0.0140 (10)	0.0044 (8)	0.0055 (8)	0.0018 (8)
O3	0.0124 (10)	0.0121 (9)	0.0149 (11)	0.0020 (7)	0.0056 (7)	0.0010 (8)
O4	0.0397 (15)	0.0405 (14)	0.0263 (14)	−0.0019 (12)	0.0025 (11)	−0.0087 (11)
N1	0.0130 (11)	0.0143 (12)	0.0130 (12)	−0.0004 (9)	0.0021 (9)	0.0018 (9)
N2	0.0134 (12)	0.0112 (11)	0.0139 (12)	−0.0014 (8)	0.0029 (9)	0.0008 (9)
N3	0.0229 (14)	0.0270 (15)	0.0244 (15)	0.0009 (11)	0.0022 (11)	0.0015 (11)
C1	0.0159 (14)	0.0142 (14)	0.0089 (14)	0.0031 (10)	0.0017 (11)	0.0045 (11)
C2	0.0246 (15)	0.0183 (15)	0.0134 (15)	−0.0006 (12)	0.0046 (12)	0.0006 (12)
C3	0.0320 (18)	0.0206 (15)	0.0130 (16)	0.0014 (12)	0.0066 (13)	−0.0017 (12)
C4	0.0264 (16)	0.0239 (16)	0.0167 (16)	0.0065 (12)	0.0118 (13)	0.0009 (12)
C5	0.0198 (15)	0.0237 (16)	0.0204 (16)	0.0039 (12)	0.0093 (12)	0.0028 (13)
C6	0.0167 (14)	0.0174 (14)	0.0119 (15)	0.0020 (11)	0.0039 (11)	0.0032 (11)
C7	0.0137 (13)	0.0146 (14)	0.0196 (15)	0.0009 (10)	0.0056 (11)	0.0047 (11)
C8	0.0193 (15)	0.0140 (14)	0.0260 (17)	−0.0047 (11)	0.0102 (13)	−0.0008 (12)
C9	0.0217 (16)	0.0279 (17)	0.0207 (16)	−0.0119 (13)	0.0069 (13)	−0.0075 (13)
C10	0.0154 (14)	0.0169 (15)	0.0174 (15)	−0.0032 (11)	0.0001 (11)	−0.0018 (12)
C11	0.0235 (15)	0.0098 (13)	0.0112 (14)	−0.0026 (11)	0.0051 (11)	0.0007 (11)
C12	0.0198 (14)	0.0099 (13)	0.0165 (15)	0.0010 (10)	0.0075 (11)	0.0019 (11)
C13	0.0243 (16)	0.0184 (14)	0.0188 (16)	0.0003 (12)	0.0088 (12)	0.0000 (12)
C14	0.0219 (16)	0.0235 (17)	0.0309 (19)	0.0037 (12)	0.0184 (14)	0.0025 (13)
C15	0.0142 (14)	0.0189 (15)	0.0306 (18)	0.0020 (11)	0.0070 (13)	0.0021 (13)
C16	0.0191 (15)	0.0157 (15)	0.0176 (15)	−0.0018 (11)	0.0057 (12)	0.0015 (12)
C17	0.0175 (14)	0.0075 (13)	0.0198 (16)	0.0008 (10)	0.0056 (12)	0.0025 (11)
C18	0.0253 (17)	0.0283 (18)	0.032 (2)	−0.0040 (13)	−0.0003 (15)	0.0003 (14)
C19	0.0254 (17)	0.0279 (17)	0.033 (2)	−0.0028 (14)	0.0044 (14)	0.0001 (15)
C20	0.037 (2)	0.039 (2)	0.031 (2)	0.0001 (16)	0.0084 (15)	−0.0057 (16)

### Geometric parameters (Å, °)

**Table:**

Fe1—O3^i^	1.8163 (18)	C6—C7	1.447 (4)
Fe1—O3	1.8186 (19)	C7—H7A	0.9300
Fe1—O1	1.9040 (19)	C8—C9	1.521 (4)
Fe1—O2	1.9334 (19)	C8—H8A	0.9700
Fe1—N2	2.015 (2)	C8—H8B	0.9700
Fe1—N1	2.069 (2)	C9—C10	1.546 (4)
Fe1—Fe1^i^	2.7337 (9)	C9—H9A	0.9700
O1—C1	1.321 (3)	C9—H9B	0.9700
O2—C17	1.327 (3)	C10—H10A	0.9700
O3—Fe1^i^	1.8163 (18)	C10—H10B	0.9700
O3—H3B	0.8500	C11—C12	1.447 (4)
O4—C18	1.241 (4)	C11—H11A	0.9300
N1—C7	1.285 (4)	C12—C13	1.405 (4)
N1—C8	1.471 (4)	C12—C17	1.422 (4)
N2—C11	1.281 (4)	C13—C14	1.369 (4)
N2—C10	1.465 (4)	C13—H13A	0.9300
N3—C18	1.328 (4)	C14—C15	1.407 (5)
N3—C20	1.449 (4)	C14—H14A	0.9300
N3—C19	1.458 (4)	C15—C16	1.386 (4)
C1—C2	1.412 (4)	C15—H15A	0.9300
C1—C6	1.417 (4)	C16—C17	1.412 (4)
C2—C3	1.384 (4)	C16—H16A	0.9300
C2—H2A	0.9300	C18—H18A	0.9300
C3—C4	1.395 (5)	C19—H19A	0.9600
C3—H3A	0.9300	C19—H19B	0.9600
C4—C5	1.389 (4)	C19—H19C	0.9600
C4—H4A	0.9300	C20—H20A	0.9600
C5—C6	1.416 (4)	C20—H20B	0.9600
C5—H5A	0.9300	C20—H20C	0.9600
			
O3^i^—Fe1—O3	82.46 (9)	C6—C7—H7A	117.5
O3^i^—Fe1—O1	95.22 (8)	N1—C8—C9	111.9 (2)
O3—Fe1—O1	94.09 (8)	N1—C8—H8A	109.2
O3^i^—Fe1—O2	91.93 (8)	C9—C8—H8A	109.2
O3—Fe1—O2	174.23 (8)	N1—C8—H8B	109.2
O1—Fe1—O2	87.73 (9)	C9—C8—H8B	109.2
O3^i^—Fe1—N2	93.36 (9)	H8A—C8—H8B	107.9
O3—Fe1—N2	92.70 (9)	C8—C9—C10	113.9 (2)
O1—Fe1—N2	169.68 (9)	C8—C9—H9A	108.8
O2—Fe1—N2	86.26 (9)	C10—C9—H9A	108.8
O3^i^—Fe1—N1	172.13 (9)	C8—C9—H9B	108.8
O3—Fe1—N1	89.95 (9)	C10—C9—H9B	108.8
O1—Fe1—N1	87.35 (9)	H9A—C9—H9B	107.7
O2—Fe1—N1	95.61 (9)	N2—C10—C9	110.9 (2)
N2—Fe1—N1	84.90 (9)	N2—C10—H10A	109.5
O3^i^—Fe1—Fe1^i^	41.26 (6)	C9—C10—H10A	109.5
O3—Fe1—Fe1^i^	41.20 (6)	N2—C10—H10B	109.5
O1—Fe1—Fe1^i^	96.19 (6)	C9—C10—H10B	109.5
O2—Fe1—Fe1^i^	133.18 (6)	H10A—C10—H10B	108.0
N2—Fe1—Fe1^i^	94.03 (7)	N2—C11—C12	124.7 (3)
N1—Fe1—Fe1^i^	131.11 (7)	N2—C11—H11A	117.6
C1—O1—Fe1	124.69 (17)	C12—C11—H11A	117.6
C17—O2—Fe1	122.47 (17)	C13—C12—C17	119.7 (3)
Fe1^i^—O3—Fe1	97.54 (9)	C13—C12—C11	119.7 (3)
Fe1^i^—O3—H3B	103.9	C17—C12—C11	120.1 (2)
Fe1—O3—H3B	140.9	C14—C13—C12	121.7 (3)
C7—N1—C8	118.6 (2)	C14—C13—H13A	119.2
C7—N1—Fe1	122.95 (19)	C12—C13—H13A	119.2
C8—N1—Fe1	118.25 (17)	C13—C14—C15	118.8 (3)
C11—N2—C10	119.5 (2)	C13—C14—H14A	120.6
C11—N2—Fe1	124.5 (2)	C15—C14—H14A	120.6
C10—N2—Fe1	115.99 (17)	C16—C15—C14	121.2 (3)
C18—N3—C20	122.4 (3)	C16—C15—H15A	119.4
C18—N3—C19	120.8 (3)	C14—C15—H15A	119.4
C20—N3—C19	116.8 (3)	C15—C16—C17	120.4 (3)
O1—C1—C2	118.9 (2)	C15—C16—H16A	119.8
O1—C1—C6	123.2 (3)	C17—C16—H16A	119.8
C2—C1—C6	117.8 (2)	O2—C17—C16	119.2 (3)
C3—C2—C1	121.2 (3)	O2—C17—C12	122.6 (2)
C3—C2—H2A	119.4	C16—C17—C12	118.2 (2)
C1—C2—H2A	119.4	O4—C18—N3	125.7 (3)
C2—C3—C4	120.9 (3)	O4—C18—H18A	117.1
C2—C3—H3A	119.5	N3—C18—H18A	117.1
C4—C3—H3A	119.5	N3—C19—H19A	109.5
C3—C4—C5	119.2 (3)	N3—C19—H19B	109.5
C3—C4—H4A	120.4	H19A—C19—H19B	109.5
C5—C4—H4A	120.4	N3—C19—H19C	109.5
C4—C5—C6	120.6 (3)	H19A—C19—H19C	109.5
C4—C5—H5A	119.7	H19B—C19—H19C	109.5
C6—C5—H5A	119.7	N3—C20—H20A	109.5
C1—C6—C5	120.0 (3)	N3—C20—H20B	109.5
C1—C6—C7	121.6 (2)	H20A—C20—H20B	109.5
C5—C6—C7	118.3 (3)	N3—C20—H20C	109.5
N1—C7—C6	124.9 (3)	H20A—C20—H20C	109.5
N1—C7—H7A	117.5	H20B—C20—H20C	109.5

Symmetry codes: (i) −*x*+1, −*y*+1, −*z*.
